# A Potential Relationship among Beta-Defensins Haplotype, SOX7 Duplication and Cardiac Defects

**DOI:** 10.1371/journal.pone.0072515

**Published:** 2013-08-29

**Authors:** Fei Long, Xike Wang, Shaohai Fang, Yuejuan Xu, Kun Sun, Sun Chen, Rang Xu

**Affiliations:** 1 Scientific Research Center, Xinhua Hospital, Shanghai JiaoTong University School of Medicine, Shanghai, P.R. China; 2 Department of Pediatric Cardiology, Xinhua Hospital, Shanghai JiaoTong University School of Medicine, Shanghai, P.R. China; Tabriz University of Medical Sciences, Islamic Republic of Iran

## Abstract

**Objective:**

To determine the pathogenesis of a patient born with congenital heart defects, who had appeared normal in prenatal screening.

**Methods:**

In routine prenatal screening, G-banding was performed to analyse the karyotypes of the family and fluorescence in situ hybridization was used to investigate the 22q11.2 deletion in the fetus. After birth, the child was found to be suffering from heart defects by transthoracic echocardiography. In the following study, sequencing was used to search for potential mutations in pivotal genes. SNP-array was employed for fine mapping of the aberrant region and quantitative real-time PCR was used to confirm the results. Furthermore, other patients with a similar phenotype were screened for the same genetic variations. To compare with a control, these variations were also assessed in the general population.

**Results:**

The child and his mother each had a region that was deleted in the beta-defensin repeats, which are usually duplicated in the general population. Besides, the child carried a SOX7-gene duplication. While this duplication was not detected in his mother, it was found in two other patients with cardiac defects who also had the similar deletion in the beta-defensin repeats.

**Conclusion:**

The congenital heart defects of the child were probably caused by a SOX7-gene duplication, which may be a consequence of the partial haplotype of beta-defensin regions at 8p23.1. To our knowledge, this is the first congenital heart defect case found to have the haplotype of beta-defensin and the duplication of SOX7.

## Introduction

The prevalence of congenital heart defects (CHD) has risen over the past few years, with a conservative estimate of 0.4∼5% of live births. Many changes may lead to cardiac malformations, including chromosomal abnormalities, gene mutations, copy number variations (CNV), or expression level changes [1]. One of the CHD hotspots is at 22q11.2, a deletion (occurring 1/4000 live births) which is already included in the prenatal diagnoses of cardiovascular anomalies using fluorescence in situ hybridization (FISH) [2]–[4]. Deletions or duplications in 8p23.1, caused by some formations of recurrent genomic rearrangement with unpredictable breakpoints, not only results in developmental delays, mental retardation and hypophrenia, but also have a close relationship with CHD [5]–[7]. GATA4 (next to 8p23.1) is thought to have a direct influence in cardiac morphogenesis and be a main cause of heart defects [8].

We describe here a different potential pathogenesis of CHD. A child with CHD had normal results of karyotype (G-banding), FISH (22q11.2) in prenatal screening and many pivotal genes like GATA4. We found that the child carries a partial haplotype of the beta-defensin region and a duplication of SOX7 which is normally underestimated in diagnoses.

### Clinical Description

**Figure 1:**
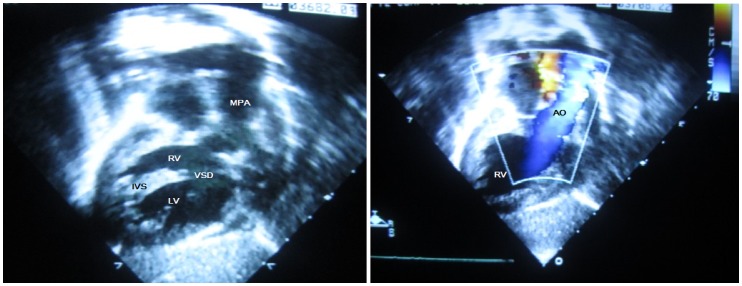
The echocardiography images of this case. MPA: main pulmonary artery, RV: right ventricular, VSD: ventricular septal defect, IVS: inter ventricular septum, LV: left ventricular, AO: aortic artery.

The child is currently a six-year-old male and was found to have a cardiac murmur during a health screening. He was diagnosed with complicated CHD when he was 4 months old. The symptoms included isolated dextrocardia, crisscross heart, double outlet of right ventricle (DORV), ventricular septal defect (VSD), atrial septal defect (ASD) and pulmonary hypertension (PH) (see Figure 1). Between the age of four months and six years old, he underwent Banding, Glenn and Fanton surgery and is under follow-up now. His mother had a cesarean section during labor due to the amniotic fluid II°contamination and the umbilical cord being wrapped around his neck. His Apar grade was 9/10 and weight was 3.6 kg. His parents were 29 when he was born; they are non-consanguineous and their karyotypes and cardiac morphology are normal. However, his sister (the proband) was diagnosed with tetralogy of Fallot (TOF) and died from anoxia when she was three years old. FISH results of the amniotic fluid were normal during his mother’s pregnancies, but she appeared to have the threat of miscarriages at early stages of these two pregnancies.

## Materials and Methods

### Subjects

Samples from the child and his parents were collected from the clinic. 50 other patients with the similar defects (DORV, VSD, ASD, PH, TOF) were recruited from June 2008 to December 2009. None of them had a definite pathogenesis. 50 unrelated healthy Chinese people (of the Han ethnicity, like the patients) were enrolled as normal controls. All samples were collected in Xinhua Hospital and Shanghai Children’s Medical Center (SCMC). Diagnoses were confirmed by transthoracic echocardiography and karyotype, and extracardiac anomalies were also evaluated. Ethics committee of Xinhua Hospital specifically approved this study, and written informed consents were obtained from the participants or their parents. The individual in this manuscript has given written informed consent (as outlined in PLOS consent form) to publish these case detail.

### Prenatal Screening: Karyotyping and FISH

The karyotypes were examined using G-banding at the 550 level. The peripheral blood (anticoagulation with heparin) and amniotic fluid were cultured in RPMI1640 culture medium with 20% calf serum (Invitrogen Gibco, USA) at 37°C in 5% CO_2_. Preparation of metaphase and conventional cytogenetics followed standard laboratory procedures. At least 20 banded metaphases with good chromosome separation were analyzed by experienced geneticists in each case. The commercially available locus specific probe kits N25 (D22S75) and TUPLE1 (HIRA) were purchased from Vysis (Downers Grove, IL, USA). Dual-color FISH was performed on metaphase spreads of the amniotic fluid cells, and 50 interphase nuclei were analyzed for the number of signals presented for each probe. Images were captured using an Olympus BX51 fluorescence microscope (Olympus, Japan). All of the screening was carried out in SCMC and followed the routine protocols [9].

### After birth screening: Sequencing, SNP-array and quantitative real-time PCR (qPCR)

Peripheral blood samples were exsanguinated into an EDTA-anticoagulate tube. DNA was extracted using the QIAamp DNA Blood Midi Kit (Qiagen, Duesseldorf, Germany) by following the manufacturer’s instructions. Purified genomic DNA was resuspended in ddH_2_O for SNP-array analysis or in Tris-EDTA for other experiments, and DNA stocks were stored at −80°C.

Pivotal genes and regions with previously associated syndrome were identified through published articles. Multiple anomalies are influenced by the genes, including TBX1, TBX5, GATA4, GATA6, NKX2.5, SOX7 and FOG2 [10]. Information about the genes was searched from Build 36.1, which was released by the National Center for Biotechnology Information (NCBI, http://www.ncbi.nlm.nih.gov/) in March 2006. The primers concerning the sequencing were designed by our group using Primer 5. 0 software and listed in Table 1. PCR products were sequenced using the Sanger method on an ABI 3130 sequencer (Applied Biosystems). The sequence traces were aligned with the reference sequence in NCBI BLAST.

**Table 1:** Primers for sequencing TBX-1, TBX-5, GATA-4, GATA-6, SOX7, Nkx2.5 and Fog2.

Gene	Primer-F (5′-3′)	Primer-R (5′-3′)
TBX1-1	GGAGGAGCAGATGTCTCAGC	CCGGCTGCCTATACTCACTC
TBX1-2	CCATGACGCCATAATCCTCT	TTGTGTTTTCTCCCCTTTGC
TBX1-3	ACGCAGCTCTCGCATTTCT	GGCGGAGGATAGGTGTTAGG
TBX1-4	GCCAAGCTCCCAGTTGAGTA	TCGCAGGTGCCTAAAGAGTT
TBX1-5	GCAGCAGAGGGTTCAATCTC	TAGCCTCGCAGGGACTCTAA
TBX1-6	AGTGACCCAGCCTCATCTTG	GTCTAAGCGGACCCACTGTC
TBX1-78	CTTGGTGCGCTTCTCCTAAC	AGAGGGCCGAGGAGTGAG
TBX1-9A	GTTGGGAGATGCAGTCCTGT	TACTGGACAGCAGCACTTGG
TBX1-9B	GATGGTGTGTGAGGCTGATG	CTTGCATGCACACTTGACCT
TBX1-9C	GGCCAAGAGCCTTCTCTCC	ACTGGGGAACCGGATACTTC
TBX5-1	CACTGAGTTATCGCATCCC	CACGAAGCCATTCTTATTT
TBX5-2	GTGGGAGCTAGTTGGATAGGC	CGAACAAGATGCGGTTTGAC
TBX5-3	CAAACTGCTCCCTCCTGT	AAGTAGATGGCAATACGCTA
TBX5-4	ACTGTGGGTTCAAGTGGT	AGGATCTATCTTTCGCTCT
TBX5-5	CCGCTTCCACGTTTCTCCAGG	TCTGAGCCTCCGCTTTCTCATCT
TBX5-6	CTCACCTGGTGCGTGAACTGAA	GGTAGAGGCAGAAAGCGACGAAAG
TBX5-7	AATGAAATCCCTGGCCCCTTTT	TTGTCCCCACCCCAGCACC
TBX5-8	GAAGTGGTGGGTCCCGTTGA	TGGAGGGAGGTGCTGGGTTG
TBX5-9	CTGGTTCAGCCACTCAGGAAATCT	CTCCAGCCTGGGTGATAGAGCA
TBX5-10A	TTGTATTCAGAATGGCGGTTAGGG	AAGTGAGCGGAGAAGTGCTGGTAG
TBX5-10B	TGCCCAGCCTAGAGGACATCAG	GGGGAGTAGCGTGAATGTGGC
TBX5-10C	CAAGGTCGCTGGATGCT	TTCGGCTTTCAGTAAACA
TBX5-10D	CCAACCTTCCAAACCTCCATCA	ACAACCTCTTCCTGTTTCCTCCAA
GATA4-1	GACTCCCACAGGCCAGTCAG	GACAAGCAAAGGCGGAGAAG
GATA4-2	ATTTGAAGCGTGGAAGAAGCAAC	CCTCGACAGGGCTCAAGACG
GATA4-3	TTGTTTCTGTGCGCTCTAG	TCTCACCCACGTAATCCC
GATA4-4	GAGTTAGGTGCCGTCACAGG	GGAAGAGGCCAGCAAAGTAG
GATA4-5	CTTAGGTGTTGCCTTCTCG	TTTGCTGGGCTCTTCATC
GATA4-6	GTTTGTCCCTGCCGCTGAT	GCTGCAAGTCCCACCCAGTA
GATA4-7A	GGTCATAGCCCTGGTTGTAT	AGGCTGTGCTGTGGTGG
GATA4-7B	CTGCATCCCTAATACCAAATC	AACCTCCCAGTGAAGACCA
GATA6-1	CCGTCCCCTCCCCACCCTCTTT	GAGATCGCGCGAGGAGGAAGCA
GATA6-2A	TGGAGGCGAGGTAGCGTGCAG	AACTGAGCAGCAGCGAGCGGG
GATA6-2B	CTGAGCCCCTTCGCACCCGAG	CCTAGGGCGGGCTGGGAGAGT
GATA6-2C	CACCTGCAGGGGTCGGGCAGT	AAACAGGGCCCGAGTGGAGCA
GATA6-3	CTACTGGGGCGCTCCGGGTGT	AGCGGGTGGGCGTTGGAACAG
GATA6-4	TGGAGAAGAAACCAGGGATGA	TGCATTCAAATTTTTCACTTGAG
GATA6-56	CGGCCGCCAAATTCTTTTA	AACCATAAAAAAATGATACCGATCT
GATA6-7	TGGCCAGGGTCAGGTCAGTGG	GAGTGGCCCAAGCGCCCAGTT
SOX7-1	CCGCTCTGAATCCTGGGCACC	ACTCCCTCCCTCCGTCCTCCTCC
SOX7-2A	AGTTAGCCATACTGGTTAATTTCTC	CCTCAGTGGGCATGTTCC
SOX7-2B	GACGGCTCCTCTGCCACTCA	CCATCTCCTGCCTATTACTCCC
SOX7-2C	CTGTGGGACCCGTTGGTGT	CATGGCCTCCTCTGCCTTGT
NKX2.5-1	GTGACACGAAACTGCTCATC	ACAACACCAGGCATCTTACA
NKX2.5-2A	AAGTCACCGTCTGTCTCCCTC	GCTCTGAACCGCATTCAAGTC
NKX2.5-2B	AAGCGCCGCAAGCTGAA	GGCCTCAATCCCTACGGTT
FOG2-1	TCATCTCCGAACGTGAATCCG	TGGGCAATAATCCCACCAACTC
FOG2-2	CGGATGTGGCATTATCT	TTACTCATGTCCCTCGA
FOG2-3	GAGGGTGTGAATGTGAAAGAG	CAAGCAGAGGTAGCACTTTGG
FOG2-4	GAGGTGGCTGCTGATAAAGTAC	GTTTCTGTCTAAATTCTGCGTAT
FOG2-5	GGTTTGGGAGATTTAGTTG	AAGATATTAGTCAAGCCACTC
FOG2-6	CATGAGAAGGTGCTATGGAC	GATGACGAGTTAGTGGGTG
FOG2-7	AATGGACAGCAGCAAAT	CTGGAGCAACAGAAGAAAC
FOG2-8A	GAAAAGGTCCCTGTCATTC	CAGGTAGGCACATCTCATAC
FOG2-8B	CTACACGCCACGACCCT	CATCTTGTTTCAGTCCACC
FOG2-8C	GCTTCCTCAAATGGGTGT	CAGAGCCTGATTATCCAAGA
FOG2-8D	GTCACAATACAGAAAAGCAT	GGTGCCATTTGGAAACTA

SNP-array (Illumina Omin1), which had an average resolution of 2. 5 kb, was performed following the manufacturer’s protocol for fine mapping of the potential aberrant regions in chromosomes. Data was analyzed using the Illumina Kayostudio software v1.3 (Build 36.1, CNV Plugin V3.0) in the recommended setting to identify only those regions larger than 75 kb comprising at least 50 contiguous markers. We deposited details of this experiment in dbVar (http://www.ncbi.nlm.nih.gov/dbvar) and got the accession number GSE48386 (NCBI GEO). The results were compared to cases in the Database of Genomic Variants (DGV, http://dgvbeta.tcag.ca/dgv/app/home ) and Online Mendelian Inheritance in Man (OMIM, http://www.ncbi.nlm.nih.gov/omim/) to distinguish common CNVs from likely causal CNVs. Segments that have a strong association with CHD were confirmed by qPCR.

Small portions of genomic DNA of healthy people were mixed to form a DNA pool serving as the normal control in qPCR. The gene COL1A1, which has few variations, was used as the control gene in qPCR. At least three selected genes in the targeted segments were searched in the UCSC Genome Browser (http://genome.ucsc.edu/). These genes were quantified to determine the copy number (CN), as were pivotal genes nearby. When the selected genes showed results different from the SNP-array, qPCR was trusted. When the same aberrant results were seen, the segments were next quantified in the genomic DNA of the parents and normal controls. They were considered to be common CNVs if detected in the normal controls. Otherwise, they were defined as potential CNVs if found to be parental, or causal CNVs if didn’t. These CNVs were inspected in Database of Chromosomal Imbalance and Phenotype in Humans using Ensembel Resources (DECIPHER, https://decipher.sanger.ac.uk/) and then verified in the samples of 50 patients we collected.

## Results

### Karyotyping, FISH and Sequencing

No obvious structural or numerical abnormalities were found on the metaphase spreads of the child and his parents by karyotype or FISH. Besides, the results of sequencing revealed no functional mutations in the coding sequences of TBX1, TBX5, GATA4, GATA6, NKX2.5, SOX7 and FOG2 genes.

### Genotyping and CN Determination

**Figure 2:**
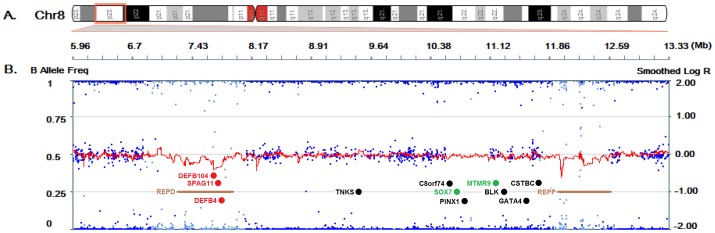
The copy number analysis of chromosome 8. (A) Ideogram of chromosome 8. (B) Results of SNP-array integrated with CNV probes. Blue spots, B allele freq; Red line, smoothed Log R; Genes were annotated: Red, deletion; Black, normal; Green, duplication. Regions of REPD and REPP were annotated in brown bar.

SNP-array showed us the child carried only one copy in each beta-defensin gene cluster (DEFB) at chr8∶7230125–7342754 and chr8∶7677945–7835713. Another deletion was found in the olfactory receptor (OR) gene cluster (chr8∶11971611–12054845 and chr8∶12273531–12392405), which is much smaller and has few genes (see Figure 2).

QPCR was used to verify the CNs of DEFB104, SPAG11 and DEFB4 in the DEFB deletion. Given that chromosomal rearrangements frequently occur in this segment and likely affect adjacent segments, verification of genes like SOX7 and GATA4 is necessary. Interestingly, the CN of GATA4 was normal, while the CN of SOX7 was 2.5 times higher than that of the normal controls. Another primer-pair designed to quantify SOX7 CN showed the same result. Verification of some other nearby genes (TNKS, C8orf74, PINX1 for SOX7 and MTMR9, BLK, CSTBC for GATA4) to make the regions distinct in the child. They turned out to be normal except for MTMR9 which has no obvious relationship to heart defects [11]–[13]. Primers’ efficiencies in qPCR were tested qualified and the sequences were listed in Table 2. We detected the abnormal loci (SOX7, MTMR9, DEFB clusters, GATA4) in the child’s parents and other 100 samples (50 patients and 50 controls). The results showed that the deletion existed in the parents. Moreover, In other 2 patients there are duplications (SOX7) and deletions (DEFB clusters). Other ratios were normal, see Table 3.

**Table 2:** Primers of the genes in qPCR.

Gene	Primer-F (5′-3′)	Primer-R (5′-3′)
COL1A1	GGGGGAACAAGGCTGTCT	TCCTGGGGTTCAGACCAA
DEFB104	AGCATTCTCTATCCCCCTCC	CATGCATAGGTGTTGGGACA
SPAG11	AGAAGTCATCCTGGAGCACA	GTGACGGACGGGAGCAAT
DEFB4	AGTTCTTACACGCTGTTTGC	AATCCGCATCAGCCACA
TNKS	TCAAAGCAAACCCATATTTTACTC	GCCAGTTAAAATAAAGCCATGTAG
C8orf74	TCGCCATCTTGGACCTGA	TTTCCTTTGCTCGCTCTTT
SOX7	GGGACATGGATCGCAATGAA	CAGCCAGGACGGAGATGAGG
SOX7-2	GCGACTCTGGACAAGTCACATC	TTATCTCACCGAATCTTCACAACA
PINX1	AGGTTCCAGTTCCAGGGTC	TTTGGGCTTCAGGGTGA
MTMR9	CACCAAGCAGAAGTGGGAGG	TGCCCAGAAATGTTCCAAAC
BLK	CTGCTCATGGTCCTTCCTC	TTGGCAATGCTTCAGTGGT
GATA4	GACATAATCACTGCGTAATCTTC	CTCCCTCCAGTCCCATCA
CSTBC	ATAACCAAGATGCTACC	CTTCTAGTTTGCTCTATACC

**Table 3:** CNs of genes in the family, other patients and normal controls.

Gene	Child	Mother	Father	Patient 1	Patient 2	Others
DEFB104	0.35	0.58	0.76	0.59	0.67	n
SPAG11	0.37	0.49	0.75	0.54	0.65	n
DEFB4	0.34	0.53	0.72	0.58	0.67	n
TNKS	n	n	n	n	n	\
C8orf74	n	n	n	n	n	\
SOX7	2.5	n	n	1.53	1.64	n
PINX1	n	n	n	n	n	\
MTMR9	1.52	n	n	n	n	n
BLK	n	n	n	n	n	\
GATA4	n	n	n	n	n	n
CSTBC	n	n	n	n	n	\

Left: Genes we detected. Right: CNs of the family, two patients, and other samples(50 controls and 48 patients); n: normal ratio comparing with the DNA pool. \: no data.

## Discussion

### The Repeat Regions

Many antimicrobial beta-defensin genes are located at 8p23.1, such as DEFB104, DEFB105, and DEFB106. The DEFB clusters are polymorphic in CN from 2 to 12 (4 is the average in the Chinese population), and the single copy has a frequency of 0. 2%∼0. 7% [14]. Rare report of single copy means that this CN haplotype may induce fatal diseases in early development [14]–[17]. Individual DEFB CN has been suggested as a genetic risk factor for psoriasis, ANCA-associated small vasculitis, Crohn’s disease and prostate cancer [5], [18], [19]. Two DEFB clusters, at 7.16–7.39 Mb and 7.67–7.89 Mb respectively, were separated by a gap containing the olfactory receptor (OR) gene cluster. The whole region is collectively named the REPD (distal repeat), and at a distance of 4.7 Mb away is another smaller repeat unit, the REPP (proximal repeat) [14], [16], [20]–[22]. S. Giglio reported that haploinsufficiency of the region between WI-8372 (6.36–6.57 Mb) and D8S1825 (8.86–9.06 Mb) was associated with congenital heart defects using short-tandem repeat (STR) analysis [23], which suggested that the REPD may be related to CHD. However, Chen found that the microdeletion (chr8∶7227000–7916187) was insufficient to induce heart defects [24].

### Recombination and Heart Defects

Florida reported some kinds of 8p23.1 nonrandom recombination that was consistently of maternal origin [25]. For three of the described recombinations, inv dup (8p), der (8p) and del (8p), Giglio considered REPD and REPP as the substrates for the formation of non-allelic homologous recombinations. Common 8p23 polymorphic inversion has a frequency in general populations of 25.6% in European [21] and 34% in Japanese [22]. However, inv dup (8p) was suggested as an independent risk factor for abnormal recombinations, which lead to several diseases, including mental retardation, facial dysmorphisms, brain defects and some other syndromes [7], [25]–[27]. Studies mentioning heart defects, always refer to the transcription factors SOX7 and GATA4, which are located between REPD and REPP in the polymorphic inversion region. Both deletion and duplication of these genes are associated with recombinations [28]–[31]. That is to say, the recombinations would highly increase the mutation risk of SOX7 and/or GATA4, thereby inducing heart defects [21]. In clinic, when a patient with heart defects due to abnormal 8p23 was found to be carrying a duplication of SOX7 and GATA4, the dosage of GATA4 was always defined as likely the most responsible cause [32], [33]. While SOX7 alterations have been seldom reported in patients with heart defects, SOX7 is a tumor suppressor in many organs [34], [35]. Whether CN variation of SOX7 (without changes in GATA4) leads to heart defects in patients needs to be further studied.

### SOX7 in Heart Development

SOX7, which belongs to the family of proteins equipped with SRY-type HMG boxes, was first identified in Xenopus and in mouse [36], [37]. SOX7 has the ability to select, bind and bend DNA chains, and interact with partner proteins (MEF2C, β-catenin) and growth factor signaling pathways (VEGFs). Previous research demonstrated that SOX7 was widespread and played essential functions during cardiovascular development in zebrafish, frogs mice and human, while SOX18 has the same role in cardiogenesis, allowing it to substitute when SOX7 is insufficient [38], [39]. Researchers injected SOX7 RNAs into the animal caps of Xenopus cell and found it would enhance the expression of MHCa, TBX5 and regulate the Xnr genes and Nkx2.5. With the increasing of SOX7 RNAs in the later stage when SOX7 should have disappeared, the Xenopus showed much more defective embryos [40]–[44]. Besides, Wnt11 inhibits canonical Wnt signaling and acts through the protein kinase C (PKC) and jun kinase (JnK) to induce cardiogenesis [39], [40], [45].

In the experiments in mouse embryonic stem cells, SOX7 was found to dictate cell fate of cardiovascular progenitors: Flk-1^+^ progenitors with increased expression levels of SOX7 are associated with a vascular phenotype. However, Flk-1^+^ progenitors with decreased expression levels of SOX7 are associated with a cardiogenic phenotype [46], [47]. Some researches directly pointed out that SOX7 is only transiently expressed at the onset of hematopoietic differentiation, and the sustained expression will block the specification and maturity [48], [49]. In addition, researches in which SOX7 expression vectors were transferred to the Human embryonic stem cells proved that SOX7 over-expression will up-regulated the expression of GATA4 and GATA6 [50], [51]. Besides, with the rising expression of SOX7, cardiac differentiation would be significantly reduced [52]. SOX7 over-expression was normally described as a destroyer in the differentiations of cells or model animals but without clinic records. This is mainly because suitable specimens were difficult to find, likely because SOX7 expresses at a very early stage before we can detect it, and serious neonatal defects can rise from SOX7 up-regulation.

### Our Case

The abnormal region in the child’s chromosome 8 is a deletion of repeat regions (1 copy) and duplication of SOX7 (5 copies). The small regions and only symptom suggest the specific loci related to heart development.

1. Repeat regions: When compared to the normal controls, the DEFB CNs in this family showed much lower ratios of 1/3 (child) and 1/2 (mother) and 2/3 (father). That means the CN of the parents are as low as 2-0 and 3-1. As a result, the child carries only one copy (1-0), which has never been previously reported in the Chinese population [14]. DEFB CNs in two other patients were also lower than ordinary, at 1/2 and 3/5, respectively. Although this is not enough evidence to argue the DEFB clusters are the causes of CHD, it is noticeable that 94.4% of patients carrying interstitial deletions have cardiac-malformation children [53]. The loss of the entire REPP and most of the REPD means that recombinations happened before. REPP (Losing or gain) in DGV may won’t lead to diseases, while recombinations in DECIPHER usually include large segments of duplication or deletion which have brought about many diseases (Table 4). Unusually, the affected range in our case was so limited (shorter than 65 kb) that it was not detected by SNP-array and may cause just the symptom of heart defects.
2. SOX7: When defects are found, the stage of heart development usually has passed already. As it is difficult to detect the precise time of expression, we have to analyze genotype-phenotype correlations. The symptoms of the child (DROV, ASD, VSD, PH, etc.), his elder sister (TOF) and another patient (TOF) may due primarily to three genes located downstream of SOX7 (GATA4, TBX5, Nkx2. 5), but only GATA4 can cause all of the symptoms [10], [53]–[55]. Without an abnormality in GATA4 (sequence and CN), SOX7 became the most likely candidate gene in our case. Duplication of SOX7 alone did not exist in the 50 healthy normal controls, the general population in DGV and patients in DECIPHER. However, it is the only common abnormality in the child and two patients when REPP seems merely has the potential to impact the recombinations (Table 4).
3. Prenatal screening: Since screening for CHD will improve the quality of public health services and reduce health care costs, it has been taken seriously in many countries [56]–[58]. The most common methods of routine CHD prenatal screening, including echocardiographic examination, may reduce the incidence of CHD but still leave the pathogenesis unexplained [59], [60]. Normal karyotype and negative results of FISH make genetic counseling difficult. Prenatal molecular diagnosis for CHD mainly aimed at mutations of the pivotal genes (such as GATA4, GATA6, TBX5 and so on) and CNVs of 22q11. Some MLPA kits (http://www.mlpa.com/) were launched to detect the CNVs of the genes and chromosomes (but without any SOX7 probes) [9], [61]. CGH array and SNP array have also been performed as a comprehensive method to analyze the genetics of heart defects, but the probe numbers and false negative results are issues that should be taken seriously. In our study, however, we found three patients with SOX7 duplication in an average-sized-patient group. That there are few reports about SOX7 duplication in CHD patients may be because of being overlooked in diagnosis. Copy number of SOX7, as a single locus for CHD and a reference locus for GATA4, has the potential to be a potential hotspot in the future.

**Table 4:** Comparison among the subjects’ genotypes and symptoms among samples.

Sample	Cardiac defects	REPD	SOX7	GATA4
Father	None	−	n	n
Mother	None	−	n	n
Child	DORV,ASD,VSD,PS,etc	−	+	n
patient 1	TOF	−	+	n
patient 2	SV	−	+	n
general people in DGV	none	−/+	n	n
patients in DECIPHER	ASD,VSD,PS,etc	−/+	−/+	−/+

+: duplication, −: deletion, n: normal. Losing or gain in the whole segments leads to diseases. REPD variations may not leads to diseases but impacts SOX7 which is related to CHD.

## Conclusion

Our study suggests that the loss of REPD or nearby regions may raise the risk of heart diseases by impacting the following genes, such as SOX7. To the best of our knowledge, we provide the first published evidence that the duplication of SOX7 has a strong association with heart defects using clinical specimens. Further research should be carried out to clarify and confirm the SOX7-CHD mechanism. Updated guidelines including SOX7 are needed for heart defects in prenatal screening.
